# Reaching the unreached through trained and skilled birth attendants in Ethiopia: a cluster randomized controlled trial study protocol

**DOI:** 10.1186/s12913-017-2041-6

**Published:** 2017-01-26

**Authors:** Taddese Alemu Zerfu, Henok Taddese, Tariku Nigatu, Girma Tenkolu, Joshua P. Vogel, Dina Khan-Neelofur, Sibhatu Biadgilign, Amare Deribew

**Affiliations:** 1 Addis Ababa University, College of Natural Sciences, Centre for Food Sciences and Nutrition, Addis Ababa, Ethiopia; 2 Friedman School of Nutrition Science and Policy, Tufts University, Boston, MA USA; 3 Dilla University, College of Medical Sciences, Dilla, Ethiopia; 4 University of Gondar, Institute of Public Heath, Gonder, Ethiopia; 5 Addis Ababa University, College of Health Sciences, School of Public Health, Addis Ababa, Ethiopia; 6 UNDP/UNFPA/UNICEF/WHO/World Bank Special Programme of Research, Development and Research Training in Human Reproduction (HRP), Department of Reproductive Health and Research, World Health Organization, Avenue Appia 20, Geneva, Switzerland; 7 Public Health Research Consultant, Addis Ababa, Ethiopia; 8 St. Paul Millennium Medical College, Addis Ababa, Ethiopia

**Keywords:** Cluster randomized trial, Skilled birth attendance, CORN, Intervention

## Abstract

**Background:**

Despite improvements since 1990 to 2014, maternal mortality ratio (MMR) remains high in Ethiopia. One of the key drivers of maternal mortality in Ethiopia is the very low coverage of Skilled Birth attendance (SBA) in rural Ethiopia. This cluster randomized trial piloted an innovative approach of deploying trained community reproductive nurses (CORN) to hard to reach/unreachable rural Ethiopia to improve the coverage of SBA.

**Methods:**

We used a three-arm cluster randomized trial to test the effect of deploying CORN in rural communities in South Ethiopia to improve SBA and other maternal health indicators. A total of 282 villages/clusters (94 from each arm) were randomly selected in the three districts of the zone for the study. The intervention was implemented in four consecutive phases that aimed at of provision of essential maternal, neonatal and child health (MNCH) services mainly focusing on SBA. The CORN were trained and deployed in health centres (arm 1) and in the community/health posts (arm2). A third arm (arm 3) consisting control villages without the intervention. A baseline and end line assessment was conducted to compare the difference in the proportion of SBA and other MNCH service uptake across the three arms Data was entered into computer, edited, cleaned, and analyzed using Epi-data statistical software. The presentation followed the Consolidated Standards of Reporting Trials (CONSORT) statement guidelines for cluster-randomized trials.

**Discussion:**

This trial is designed to test the impact of an innovative and newly designed means of distribution for the national health extension program strategy with additional service package with no change to the target population. The focus is on effect of CORN in revitalizing the Health Extension Program (HEP) through improving SBA service uptake and other maternal health service uptake indicators. The study findings may guide national policy to strengthen and shape the already existing HEP that has certain limitations to improve maternal health indicators. The competency based training methodology could provide feedback for health science colleges to improve the national nursing or midwifery training curriculum.

**Trial registration:**

clinicaltrails.gov NCT02501252 dated on July 14, 2015.

## Background

Motherhood in many parts of the world remains unsafe even 25 years after the launch of Safe Motherhood Initiative in Nairobi, Kenya [1]. About 287,000 maternal and 3.1 million neonatal avoidable deaths occur annually [2]. The rate of death of a woman during pregnancy, childbirth and the puerperium has shown its divergence in the poor and the rich countries [3]. Particularly, there has been less than satisfactory progress in sub-Saharan Africa, towards the global child and maternal mortality targets [3, 4].

Ethiopia has shown remarkable progress in the reduction of maternal and child mortality rates during the past 25 years [5–7]. Nevertheless, maternal mortality rates is still high in Ethiopia [8, 9]. The risk factors of maternal mortality in Ethiopia are complex and interwoven [10, 11]. However, the key factors attributable for the death of mothers are related to low facility-based deliveries, poor competence of providers, and poor quality of care and referral system [12–14]. In rural parts of Ethiopia, which accounts for 85% of the national population, delivery attended by qualified health professionals (SBA) is only 16% and 84% of deliveries occur at home [15, 16].

Ethiopia has implemented an innovative community-based health service delivery called HEP since 2003 to improve access to health care services particularly for mothers and children [17]. HEP involves trained and salaried female health cadres who provide basic primary health care services at community level. Two Health Extension Workers (HEWs) are assigned per health post for about 5000 population at *Kebele* (lowest administrative unit) level. The HEP includes 16 essential health packages under four major program areas: hygiene and environmental sanitation, disease prevention and control, and family health services and health promotion and communication [17, 18].

The HEP has contributed substantially to the improvement in women’s utilization of family planning, antenatal care (ANC) and HIV testing. However, its contribution to improve health facility delivery, postnatal care and use of iodized salt has been insignificant [19, 20]. The HEW are overwhelmed with several preventive activities including family planning, vaccination, sanitation and treatment and referral of malaria and other diseases and have very limited time and skills to improve SBA [21]. HEW is expected to identify, counsel and refer high-risk pregnancies. However, an evaluation report of the HEP showed that less than 50% of the HEW knew signs of obstructed labor [22].

Thus, to end with these alarming and potential limitations of the national HEP, an innovative and culturally sensitive strategy at community level is needed. Therefore, we conducted a cluster randomized trial to pilot the effect of deploying trained CORN to provide SBA and other maternity care service in rural Ethiopia. In this paper, we report the result of the trial in detail.

### Objectives of the trial

The trial aims to test the effect and acceptability of CORN on maternal and neonatal health service uptake in rural south Ethiopia. The specific objectives include:

1. To determine the effectiveness of deploying trained nurses to rural villages on SBA services utilization (various options).
2. To examine the programmatic and socio-cultural acceptability of home based delivery of SBA services.
3. To determine the effect of the CORN on uptake and utilization of other key maternal, neonatal and child health care services: focused antenatal care (FANC), long and permanent acting family planning methods, and postnatal care and nutrition services.
4. To assess the overall effect of CORN project in strengthening the rural health extension program.

## Methods/Design

### Settings

The study was conducted in three districts of Gedeo Zone in Southern, Nations, Nationalities and Peoples (SNNP) region of Ethiopia. The SNNP Region is one of the nine Regions in the country, which makes 20% of the national population. Gedeo Zone is one of the highly populated areas in Ethiopia [23]. The Zonal town, Dilla, is located 365 Km away from Addis Ababa, the capital of Ethiopia.

**Fig. 1:**
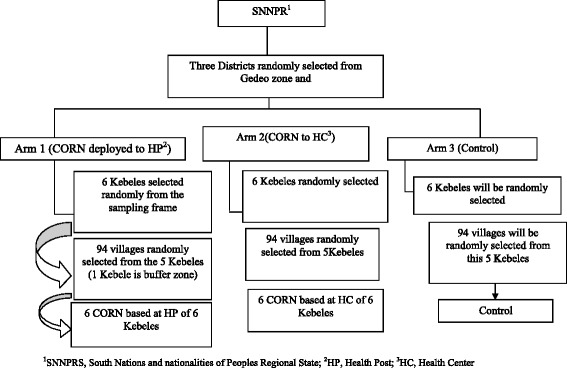
The schematic diagram of sampling procedure

Three districts namely Wonago, Yirgachefe and Kochere with population of 284,450, 320,012 and 187,654 were included in this study. The selection of *Kebeles* in each district is described below (Fig. 1). In the selected Kebeles, there are 18 health centres, and 82 health posts that provide maternal and child health care services. Dilla university hospital is a referral centre for the catchment population. The health care system of Ethiopia is a three-tier health care delivery system composed of district health system, general hospital, and specialized/teaching hospital [22]. The district health system is made up of a primary hospital (serving 60,000 populations), a health center (serving 15–25,000 population) and health posts (one health post serving 3–5000 Population).

### Design, study participants and sampling procedure

We used a three-arm cluster randomized trial to assess the effect of deploying CORN to the rural community on SBA and other maternity health care services. The trial was conducted during October 2014 to June 2016. CORN were trained in a similar setting and were randomly assigned into one of the following three arms by the investigators: **Arm one** consists of villages served by CORN based at the health centres (HC) level. The CORN at the HC level provides skilled birth attendance and other RH services on demand at household levels on an outreach basis. **Arm two** includes villages served by CORN based at health posts in the community. CORNS in arm 2 provide SBA at the health posts and home. The **third arm** consists of control villages with no CORN deployed. The HEW perform their routine activities in all the villages in the three arms.

The study included 94 villages/clusters in each arm. We used the following equation [24] to calculate the sample size (number of villages).

$$ N=2 x{\left(\frac{Z_{1-\alpha}+{Z}_{1-\beta}}{d-{\delta}_0}\right)}^2 x P x\left(1- P\right) $$

Where N is the number of villages; Zα/2 the standard normal variable at α = 5%; Zβ is (1-β) = 80%;

P is the proportion of SBA in the control villages (4%) [25]; p0 is the proportion of SBA in the interventions villages (62%) that is in line with the government ambitious strategy [26]; d is the real difference between two treatment effect (58%); and δ is a clinically acceptable margin assumed to be 50%.

In the Ethiopian context, a village is the lowest sub-administrative unit within *Kebeles* having a size of 30 to 35 households and 180 to 200 individuals. Pregnant women constitutes 4% of the total population [23] and this yields about 7 to 8 pregnant mothers per village per year. In the 94 villages in each arm there were on average 705 pregnant women. Hence, we enrolled a total of 1974–2256 (mean of 2115) pregnant women for the whole study.

We used a two-stage cluster sampling technique to select the study villages. In the first stage, 18 *Kebeles* from the three *Woredas* (districts) were selected randomly. In the second stage, 282 villages were randomly selected from a list of villages (sampling frame) obtained from the HEW. A buffer zone of *villages* between study arms were left to avoid contamination of information and overburdening of the CORN. The sampling procedure is depicted in Fig. 1.

All pregnant women who reside in the study villages and were willing to take part in the study were included. Non-resident pregnant women or resident pregnant women who were not willing to participate were excluded from the study.

### The intervention and data collection procedures

The study has preparatory, intervention and evaluation phases. In the preparatory phase, sensitization workshop with relevant stakeholders such as community representatives, ministry of health (MOH) and regional health bureau (RHB) experts in reproductive health and other were conducted. During this phase, 16 CORN were recruited through nationwide advertisement and entrance exams. The CORNS were trained based on the core competencies of the intervention. Diploma nurses or midwives (10 + 3 graduates) who had at least 48 h theoretical and 72 h practical sessions on reproductive, maternal, newborn and child health in the pre-service training (college) were eligible for CORN. The CORN were trained for 4 months at Dilla University hospital based on competency model [27]. The training focuses on key components such as practical skills, problem solving, critical thinking and decision-making skills. The 4 months training topics include basic concepts of maternal and newborn health; rapid initial assessment and managing emergency; pregnancy care; childbirth care and postnatal maternal care. In addition, they were trained for an additional month on family planning, HIV counselling and testing and community mobilization skills. In general, the training aimed to achieve the following goals: promotion of health and prevention of diseases; detection of existing diseases and treatment; early detection and management of complication and birth preparedness and complication readiness. Finally, baseline survey in all arms was conducted to assess the coverage SBA and other maternal indicators during the preparatory phase.

**Fig. 2:**
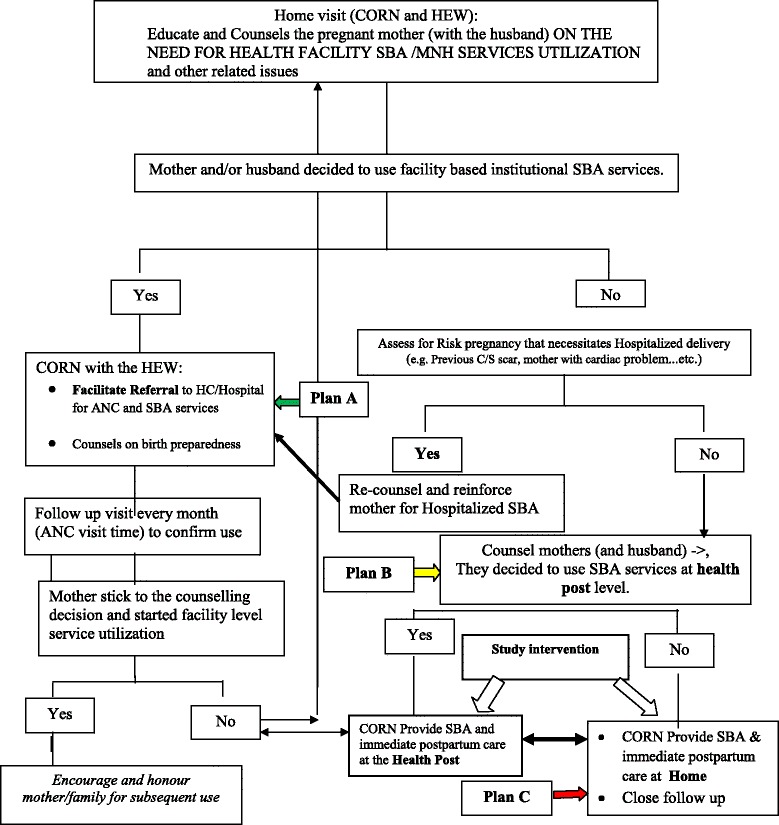
CORN Activities (Intervention) Algorithm

After the completion of all the preparatory activities, the CORN were randomly deployed to the interventions villages, either at the health centre (arm1) or community/health post level (arm 2) for ten months. The intervention algorism is described in Fig. 2. The CORN at the community level visits each household every two weeks and provides essential services to pregnant women including counselling, birth preparedness and complication readiness and referral to the health post or health centres. The CORN at the health centres provide reproductive, maternal, neonatal and child (RMNCH) services at the centres on a daily outreach basis. One mentor (experienced midwife from Dilla University) was assigned for three [3] CORN to mentor their activities and provide feedback.

During the interventions period, data on maternity service uptake such as deliveries, family planning, postnatal care and, HIV counselling and testing were collected at the health facilities or communities by assigned experts. In the control villages, routine activities were done by HEW as per the Ethiopia national guideline procedure. The HEW were also doing their routine activities in the intervention arms. Monthly coordination meeting between the CORN, supervisors, investigators and HEW were also done to monitor program performance and provide feedback. In the evaluation phase, final survey was conducted to assess coverage of maternal indicators in all arms and identify reasons of home and intuitional deliveries.

### Study outcomes measures

The primary outcome was proportion of pregnant women who give birth by the assistance of skilled professional (% SBA) and views of stakeholders in accepting CORN intervention. The secondary outcomes included proportion of pregnant women who got focused antenatal care services, HIV counselling and testing, receive family planning services with a focus on long acting methods, attended postnatal care services and neonatal morbidity.

### Data management and statistical analysis

Data were entered into Epi-data computer software, edited, cleaned, and analyzed using Statistical Package for Social Science (SPSS 21.0, Inc., and Chicago, IL, USA) and STATA 12.0 (Stata Corporation, College Station, TX). Proportion of SBA and other maternal indicators at baseline and endline of the study and among the intervention and control villages was compared by adjusting confounding variables. Confidence intervals of percentages were calculated by taking into account the villages as a cluster variable using weighted t-tests in STATA. Multivariate analysis will be using the simultaneous entry complex surveys logistic regression model by taking into account the clustering effect at the village level. A *P*-value of less than or equal to 0.05 will be considered to be significant for all tests. The presentation has also followed the CONSORT statement guidelines for cluster randomized trials [28].

### Quality assessment/control

Reliability and validity are considered as a criterion for assessing the quality the study. For this fact, randomization list was kept confidential on the study participants’ assignment to which group. A written consent was obtained from the participants before the study enrollment. The supervisors, principal investigator and data collectors will meet regularly in the field and at Dilla University. The data collectors, local supervisors and data monitoring coordinator checked the completeness and consistence of collected quantitative data. Then the principal investigator and research team analyzed the final dataset. Additionally, data collector guaranteed quality via systematic observations of data collection and confirmation was done from the participant's record file at the health facilities. Loss to follow-up from the study was minimized by home visit by the research team members and supervisors. A unique Identification number was provided to the participants during participation in data collection period and data analysis.

## Discussion

The maternal mortality rate (MMR) in Ethiopia in 2013 (497 per 100,000 live births) showed that there was no significant decline over the last two decades [7]. The low coverage of SBA is the main driver of maternal mortality in Ethiopia. With this low coverage of SBA, Ethiopia will face tremendous challenge to meet the Sustainable Development Goals (SDGs) [29, 30].

The flagship HEP in Ethiopia uses a Family Folder that is a low-cost and high impact health management information system at *Kebele* level to make health services accessible to the poor [17, 31]. This program has been successful in reducing the burden of malaria [32] and improving the coverage of ANC [19]. However, the coverage of SBA remains low in the last ten years despite the presence of HEW in the community. This calls for an innovative approach to improve SBA nationally. This cluster randomized trial aims to assess the impact of such an innovative approach of deploying CORN to rural Ethiopia on the coverage of SBA. The findings and lesson learnt from this trial will guide national policy to design need-based and relevant alternative interventions to improve SBA in rural Ethiopia. The study may also guide policy makers how to scale up other maternal interventions such as the Prevention of Mother-To-Child Transmission (PMTCT) during the SDGs era. The study findings would also highlight the effectiveness of such an innovative approach on maternal health indictors. More importantly, the lesson learnt from this study may provide feedback to Universities to improve the curriculum of nurses and midwives nationally.

The study used a rigorous study design to answer the research question. Buffer *Kebeles* were also left to reduce contamination of the intervention. However, contamination of information may not be controlled totally. Furthermore, as the study is innovative by nature whereby nurses deployed to work in rural villages where there is little or no infrastructure including food, there is a possibility of in and out stay on job. The nurses usually travel to nearby towns to fetch water and bring food that might affect full implementation of the interventions throughout the project stay. Another possible limitation is through agreed to support and were smoothly working with the project staff, there is still a possibility of discouraging home based skilled delivery as it is not a reportable activity and fear of community's experience with home based skilled delivery.

## Conclusions

The study design is relevant and rigorous to assess the impact of deploying CORN on coverage of SBA in rural Ethiopia. The study findings may guide national policy to implement relevant interventions to meet the SDG goals. The competency based training methodology could be helpful to improve the national nursing and midwifery training curriculum.

## Abbreviations

ANC: Antenatal care
CONSORT: Consolidated Standards of Reporting Trials
CORN: Community reproductive nurse
HEP: Health extension program
HEW: Health extension workers
MMR: Maternal mortality rate
PMTCT: Prevention of Mother-To-Child Transmission
SBA: Skilled birth attendance
SDG: Sustainable development goals
SNNP: Southern, Nations, Nationalities People Region
WHO: World Health Organization
